# Resilience and Coping with Stress and Marital Satisfaction of the Parents of Children with ASD during the COVID-19 Pandemic

**DOI:** 10.3390/ijerph191912372

**Published:** 2022-09-28

**Authors:** Anna Gagat-Matuła

**Affiliations:** Institute of Special Needs Education, School and Teachers Education, Pedagogical University of Cracow, 30-084 Cracow, Poland; anna3006@op.pl; Tel.: +48-050-911-7137

**Keywords:** marriage, satisfaction, resilience, coping styles, autism spectrum disorders, COVID-19 pandemic

## Abstract

Raising and caring for a child with ASD is a challenge for the parents’ marriage relationship. Caring for a disabled child changes the functioning of the family and requires greater involvement in care and specialist therapy. The respondents’ answers show that such parents experience higher levels of stress related to the upbringing and future of the child. However, parenting challenges need not be a negative experience if the parents support each other. The process of bringing up children in the family are related, inter alia, to the quality of their parents ‘marriage, which is influenced by the partners’ personal resources. The resilience of the partners and coping with this situation contribute to marital satisfaction. The aim of the study is to find out about the relationship between spouses’ resilience and coping styles and their assessment of marriage satisfaction. In total, 50 married couples participated in the study—50 mothers of children with ASD and 50 fathers, the partners of these mothers (N = 100). The following tools were used: the Resilience Scale (SPP-25), the Coping Inventory for Stressful Situations (CISS) and the Well-Matched Marriage Questionnaire (KDM-2), as well as a survey questionnaire (data on respondents). The results show that the gender of the parent of a child with ASD does not differentiate the overall assessment of the quality of marriage (the overall score on the scale indicates a low level of satisfaction with the relationship). When analyzing in detail the dimensions of individual scales of satisfaction with the relationship, one statistically significant difference was noted for the sex of the respondents in the disappointment dimension, showing that the level of disappointment in the quality of the relationship is higher in wives than in husbands. In the other dimensions of satisfaction with marriage: intimacy, personal fulfillment, similarity, there were no statistically significant differences in terms of gender of the respondents. The resilience of the spouses positively correlates with their assessment of marriage satisfaction, and in particular, openness, perseverance and determination to act increase the level of Task-oriented coping (SSZ) with difficult situations. All resilience factors negatively correlate with the emotional coping style (SSE). In both the studied groups, openness is a significant predictor of intimacy, and persistence is a predictor of self-fulfilment in a relationship. A predictor of disappointment in women is managing using substitute activities (ACZ), while in men it is the Emotion-oriented coping (SSE) style. The results indicate the need to support married couples raising a child with ASD during the pandemic.

## 1. Introduction

On 11 March 2020, the World Health Organization (WHO) announced the outbreak of the COVID-19 virus as a public health emergency of international concern, identifying it as a global pandemic [1]. The resulting situation significantly affected every aspect of human functioning, threatening an individual’s life [2] and limiting their social functioning through the restrictions introduced, which over time significantly worsened people’s mental condition, leading to anxiety disorders and depression [3].

Families and people with disabilities found themselves in a particularly difficult situation. Their quality of life, including their financial situation, mental condition and progress in rehabilitation significantly worsened [4,5,6]. Research shows that the level of stress related to the pandemic is much higher among people with disabilities and their family members than among non-disabled people [7,8]. The situation of families raising a child with autism spectrum disorder (ASD) is extremely difficult due to the nature of the complexity of the disorders that occur in the child. The symptoms of the autism spectrum focus on permanent deficits in social communication and social relations in many environments, as well as limited, repetitive patterns of behaviour or activity, including the presence of numerous difficult (aggressive, autoaggressive) behaviours. The effect of autism spectrum on the population varies greatly from person to person. This can be from high-functioning to low-functioning, with high limitations and coexistence of intellectual disability, hence sufferers and their families require support to a varying extent. As already mentioned, people with low-functioning ASD have great difficulties in using speech and communicating with others. They often have intellectual disabilities, stereotypical patterns of behavior and activity, and compulsive behaviors that significantly limit functioning. Unlike people diagnosed with low-functioning autism, those with high-functioning autism are good at speech but do not understand metaphors, jokes and irony. Despite the fact that they use speech well, they have difficulties in establishing and maintaining relationships with other people, are at the intellectual norm or even have above-average intellectual abilities. They have sterotypical interests that consume their time and activity. Both people with high-functioning and low-functioning autism have great difficulty adapting to changes and are very attached to daily routine. In both cases, there may be numerous difficult behaviors (physical, verbal aggression, autoaggression), as well as the coexistence of emotional disorders. Due to the difficulties in the functioning of a person with ASD and their immediate environment, including the family, they require support [9]. Research shows that isolation during the pandemic significantly limited therapeutic services provided to families and children with ASD, forcing them to look for other solutions for the guidance and support that is so much needed [10].

The high level of stress associated with the pandemic worsened relationships in families in which a child with ASD was being brought up [11,12], especially since these families experienced high levels of stress even before the pandemic. Permanent stress translates into the quality of the marital relationship. Research shows that parents of children with ASD have lower satisfaction with their relationship than parents of non-disabled children or parents of children with other developmental disorders [13,14,15,16,17,18,19,20]. The consequence of these factors are conflicts in the relationship [21] and a higher divorce rate in the group of married couples with a child with ASD. It is worth pointing out that the rate of divorce remained high throughout childhood, adolescence and early adulthood for parents of children with ASD, whereas it decreased in childhood (after about 8 years of age) in the control group [22]. Hence, it is extremely important to assess the satisfaction with the marriage of parents of children with ASD during the pandemic.

It is worth noting that this article refers to the concept of marital satisfaction developed by Plop and Rostkowski. According to this concept, satisfaction with a relationship has a multidimensional structure, which consists of four basic dimensions:

- Intimacy—is associated with a high level of satisfaction resulting from a close relationship with one’s spouse and the assumption that the partners want to build their own relationship based on full openness, mutual trust, closeness and honesty. Thus, intimacy reflects how much the spouses feel that they are united by a love that allows them to fully accept their partner;
- self-fulfilment—this dimension means that thanks to a satisfactory marriage relationship, partners gain the opportunity to fulfil themselves following their own system of values, as well as specific life tasks;
- similarity indicates the level of agreement of the partners in the realization of essential marriage and family goals;
- disappointment, which indicates a sense of failure in life caused by the fact of getting married, as a result of which marriage is perceived and experienced as a factor that threatens the independence and autonomy of the partners [23].

Conceptualization of satisfaction with marriage leads to the question of the determinants of satisfaction of partners with their mutual interactions. In this area, the variables of each spouse are analysed—their personal resources (resilience and styles of coping with stress). In this project, resilience is understood in the category of a personality trait, a specific mechanism of self-regulation encompassing cognitive, emotional and behavioural elements [23], while coping styles are remedial actions undertaken by a person in a specific stressful situation, and are the result of the interaction between the features of the situation and the style of the individual’s coping characteristics. This style is their typical behaviour in various difficult situations. Endler and Parker distinguish between three types of styles: task-focused, focused on emotions and focused on avoidance [24].

Since marriage is a relationship that passes through various life situations, the personal resources of the spouses, which may be decisive for the successful overcoming of difficulties experienced in mutual interactions or may influence them, are of significant importance. The most important of these are resilience and styles and strategies for coping with stress [24]. Previous studies confirm and show the relationship between the personal resources of parents of children with ASD and marital satisfaction [25]. In the process of coping with stress, the quality of the marriage relationship is of great importance, including, above all, the support of the spouse [26]. Research shows that the high quality of marital relations between parents of children with ASD reduces the impact of stress and lowers the risk of depression [27,28,29,30]. Moreover, research results show that a strong bond between spouses may act as a buffer against the influence of stress factors related to caring for a child with ASD [31].

As mentioned earlier, the pandemic can be one such stress factor as it has certain consequences, such as fear for life and health, isolation, risk of loss of income, problems with access to health care, including rehabilitation, an increase in difficult behaviour in a child with ASD, and a lack of progress in therapy. This difficult situation can make parents exhausted and feel overburdened, so personal resources such as resilience and styles of coping strategies that affect marriage satisfaction are important.

Hence, the aim of this research is to find out about the relationships between the resilience and coping styles of parents of children with ASD and their assessment of marital satisfaction during the COVID-19 pandemic. The following research questions were posed:

1. Do, and if so, how do the wives and husbands studied differ in terms of resilience, coping styles and ratings of marital satisfaction?
2. If they exist, what are the relationships between resilience and coping styles in the groups of wives and husbands?
3. Are resilience and coping styles related to the assessment of marital satisfaction in the group of wives, and if so, how?
4. Are resilience and coping styles related to the assessment of marital satisfaction in the group of husbands, and if so, how?

## 2. Materials and Methods

The research was conducted in 2020 and 2021 at the Specialist Outpatient Clinic for People with Autism in Leżajsk. 50 married couples participated in the study—50% were mothers of children with ASD and 50% of fathers, the partners of these mothers. This was during the period of the COVID-19 pandemic in Poland, and related restrictions (including isolation) and access to the research group was significantly limited. The average age of the parents studied was 44 years (from 21 to 56). The majority of the women had secondary education (69%), followed by higher (20%) and vocational (11%) education. The men, on the other hand, had mostly secondary education (62%), followed by vocational (21%) and higher (17%) education. In the case of origin, a division of the studied population into three groups can be made: village—40% of respondents, city up to 100 thousand—33% of respondents, city 100–500 thousand—27% of respondents. The duration of the relationship of the respondents can be illustrated as follows: 1–10 years (66% of respondents); 11–20 years (24% of respondents). The criterion for selecting the married couples was that they had a child with autism spectrum disorder (ASD), communicating non-verbally, with auto aggressive behavior at the age of 5–8. The children had a diagnosis of autism spectrum disorder with level 3 autism spectrum disorder (according to the DSM-5). In the research, the child with ASD was most often the first child (53%), the second child in the birth order (28%), and the third (19%). Raising a low-functioning child with uncertain prognosis is a very stressful challenge for parents, and was especially during the COVID-19 pandemic, where access to specialist therapy was limited. Hence, the couples received a set of questionnaires with instructions and a request to complete and return them within the prescribed period. The respondents were assured of the anonymity and purely scientific nature of the research. Incomplete questionnaires were excluded. The research was approved by the relevant bioethics committee. The purpose of the study was explained to the participants and they were informed about its anonymity.

The following tools were used in the study: a questionnaire containing questions on general information about the respondents, i.e., gender, age, place of residence, education, years of marriage, age of the child and its functioning.

The Resilience Scale (SPP-25) by Ogińska-Bulik and Juczyński was used to measure resilience. It contains 25 items and measures the general elasticity index and the five factors that make up its structure. The respondent assesses the degree to which a given statement applies to him on a 5-point Likert scale (from 0—definitely not, to 4—definitely yes). The obtained raw numerical result (both for the general index and for individual factors) is converted into sten norms in accordance with the diagnostic key. The SPP-25 scale is characterized by good reliability for the entire scale (Cronbach’s 0.85), and the reliability indicators of the five subscales are in the range of 0.67–0.75 [32].

The Coping Inventory for was used to measure coping styles in stressful situations (CISS) by Endler and Parker, in the Polish adaptation by Strelau, Jaworska, Wrześniewski and Szczepaniak. It consists of 48 items, and the test person’s task is to respond to each statement by marking the answer on a 5-point Likert scale (from 1—never to 5—very often). The obtained numerical result was analysed in accordance with a key developed by the authors of the questionnaire and allows for the measurement of three styles of coping with stressful situations. The obtained raw results for individual scales are converted into sten norms for people in three age groups: 16–24 years, 25–54 years and 55–79 years. The CISS questionnaire is characterized by high reliability—the Cronbach values for individual scales are 0.72–0.92 [33].

The Well-Matched Marriage Questionnaire (KDM-2), by M. Plopa and J. Rostowski, was used to measure marital satisfaction. It examines the level of marital satisfaction and is intended for people who are married. The questionnaire consists of 32 statements, to which the respondent answers on a 5-point Likert scale (from A—“I completely agree” to E—“I completely disagree”). The results are calculated in four dimensions representing the level of satisfaction with the relationship: intimacy, self-fulfilment, likeness and disappointment. The sum of the scores on the individual subscales gives the overall score. The psychometric indicators of the questionnaire are satisfactory. The reliability for the various dimensions included is in the range of 0.80 to 0.89. The tested internal validity of the questionnaire was checked using the following methods: examining the internal structure of the test, and checking intergroup differences and criterion validity [23].

## 3. Results

All calculations were performed using Statistica 13 software. For each variable, the normality of the distribution was verified using the Shapiro-Wilk test, which is recommended for research groups smaller than N = 100. Analysis of the distributions of the studied variables indicated the dominance of the normal distribution. In order to check whether and how wives and husbands raising a child with ASD during the COVID-19 pandemic differ in terms of the studied variables, the Student’s *t*-test was used for independent groups due to the presence of a normal distribution among the studied variables. The raw results and the results in sten are given in Table 1 below.

The conducted comparative analysis of the average results obtained by the studied groups in the measurement of resilience and styles of coping with stress did not show statistically significant differences in the scope of the studied variables. This proves that the studied wives and husbands do not differ in terms of the level of resilience and styles of coping with stress. It is worth noting that the level of the researched resources is moderate. It is difficult to make an unequivocal conclusion on the reason for the obtained similarity of psychological resources of the surveyed spouses raising a child with ASD. Perhaps the earlier building of long-term relationships with a life partner allows for mutual similarity in terms of mental resilience and styles of coping with the hardships of living together. It cannot be ruled out that jointly struggling with a child’s disability contributes to strongly supporting one another, similarity of styles in coping with a difficult and stressful situation, coming to agreement on actions, communication and a feeling of closeness, as well as mutual trust in terms of help and support from a partner in the upbringing of a child with ASD and the therapy required.

There were also no significant statistical differences between the studied groups in terms of the perceived general level of marital quality. The mean results range from 4 to 7 sten, indicating low and moderate satisfaction with the marriage. The overall result for the respondents is low (sten 4), which indicates an unsatisfactory assessment of the quality of the marriage by the respondents. The scores for the individual scales are also low to moderate. The only significant difference among the studied wives and husbands raising a child with ASD was revealed in terms of disappointment with marriage, which was high among the wives and average among the husbands. In the wives, this may probably indicate a sense of failure in life, and may also be a signal of a disturbance in independence and personal autonomy related to overload and stress related to raising a child with ASD, especially during the COVID-19 pandemic. This may indicate that marriage, to some extent, prevents them from realizing themselves, their value system and their life tasks. In the remaining dimensions, the respondents obtained similar results, which proves a similar assessment of the quality of the marriage relationship.

Subsequently, the relationship between resilience variables and styles of coping with difficult situations by wives and husbands was established using Pearson’s r correlation coefficients (see Table 2). For this purpose, r-Pearson correlation analysis was performed, and the conditions for the correlation were met (the data interval and distribution were close to normal and were checked using the Kolmogorov-Smirnov test).

The above correlation matrix allows for the identification of many significant differences in the relationships between resilience and coping styles among the groups of wives and husbands raising a child with ASD during the COVID-19 pandemic. In the group of wives, the following resilience factors increase the level of coping with a difficult situation: perseverance, openness, competence, tolerance and overall resilience. However, there was no correlation between the wives’ optimistic attitude and their task-oriented coping style. All the factors of wives’ resilience reduce their tendency to use the emotional coping style, although only some to a statistically significant degree. These are: openness, competence, optimistic attitude, as well as general resilience. Nevertheless, in the group of wives, an increase in the level of the avoidance style is observed, manifested in the search for social contacts by the following resilience factors: openness, competence, tolerance, optimistic attitude and general resilience.

In the group of husbands, only some factors of their resilience correlate directly and proportionally to a statistically significant degree with the task-oriented style of coping with difficult situations. These are: perseverance, optimism and general resilience. Resilience lowers the level of using the emotional coping style, especially competence, tolerance, an optimistic attitude and general resilience. A similar regularity is apparent in the relationship between the husbands’ resilience and their use of the avoidance style—engaging in substitute activities, and there is a particularly strong relationship between perseverance and husbands taking substitute activities. The similarity between the two groups is found in the relationship between perseverance and general resilience and the preference for the task-oriented style in coping with stress. In turn, the differences between wives and husbands are in the relationship between resilience and the avoidance style—in women, resilience increases the search for social contacts, and in men it lowers the style of engaging in alternative activities.

Analysis was conducted of which factors of resilience and what style of coping with stress in the group of wives influenced their assessment of marital quality. The results are presented in Table 3.

In the table above, the predictors of marital satisfaction in the group of wives were distinguished. For this purpose, regression analysis was carried out with the progressive stepwise method, which showed the important role of the following resilience factors: perseverance, openness to new experiences and an optimistic attitude to life. The listed factors explain 14–35% of the variance of the dependent variable—marriage satisfaction. A detailed analysis of the impact of resilience on marital satisfaction shows that openness directly affects intimacy (14% of variance), persistence and openness to new experiences directly affect self-realization and similarity (35% and 27% of variance), while an optimistic attitude to life has the opposite percentage effect on both self-realization and likeness. Regression analysis confirmed the directly proportional impact of style of coping with difficult situations (such as engaging in substitute activities) on disappointment in the relationship. Thus, the predictors of marital satisfaction in the group of wives studied are three resilience factors: persistence and openness (acting in direct proportion), an optimistic attitude (acting inversely), and the style of coping with difficult situations, such as engaging in substitute activities to make up for disappointment in the relationship.

In the group of husbands under study, the predictors of individual dimensions of their marital satisfaction were sought, and the results obtained in the regression analysis are presented in Table 4.

In the group of husbands, regression analysis with the progressive stepwise method revealed a significant influence of two resilience factors on the assessment of marital satisfaction and two styles of coping with stress. Openness has a directly proportional effect on intimacy (32% of the variance of the dependent variable). Perseverance and determination in action show a directly proportional effect on self-actualization (33% of the variance of the dependent variable), as does similarity (22% of the variance of the dependent variable). Moreover, the predictor of disappointment is the emotional coping style of husbands (b = 0.145), while engaging in substitute activities (avoidance style) turns out to be a predictor of self-fulfilment (b = 0.144). None of the styles of coping with stress by husbands turned out to be a significant predictor for the dimensions of intimacy or similarity.

Summarizing the stepwise regression analysis in both research groups, significant differences in the identification of predictors of marital satisfaction should be emphasized. In the group of wives, the predictors of ties are three resilience factors: openness and perseverance, which act in direct proportion, and one inversely proportional—an optimistic attitude to life. One of the studied styles of coping with stress (avoidance), that is engaging in substitute activities, acted directly in proportion to disappointment—the dimension of marital satisfaction. On the other hand, in the group of husbands, the predictors of marital satisfaction are openness, persistence and determination in action, as well as the emotional and avoidance styles for coping with stress. These explanatory variables have a direct proportional impact on the dependent variable.

It can be concluded that the gender and social role (wife, husband) of the studied parents of a child with ASD during the COVID-19 pandemic are important non-psychological factors in the struggle caring for a disabled child.

## 4. Discussion of the Results

The studied spouses bringing up a child with ASD during the COVID-19 pandemic did not differ in terms of the level of resilience and the style of coping with stress. Both groups achieved an average level of resilience, optimal for constructive psychosocial functioning (5–6 sten). The spouses perceive the current pandemic reality and raising a child with ASD in terms of a challenge, and are convinced of their own competence for coping with the difficulty of raising a child with a disability, even in such a difficult period as the pandemic, when there are limited services related to the rehabilitation of the child. They use various methods of coping with difficult life situations, while maintaining emotional stability. Resilience, treated as a personality trait, protects their mental balance, reduces the likelihood of the spouse’s symptoms related to maladjustment and burnout associated with raising a child with a disability. The level of their resilience shows the respondents as people able to flexibly respond to the changing perspectives of a child’s rehabilitation during the pandemic, temporary isolation and the associated difficulties. The results they obtained, indicate an optimal level of resilience (with the most pronounced being persistence and determination in action). This is confirmed by previous studies conducted before the COVID-19 pandemic, where parents had moderate levels of resilience, which indicates that they are resistant to adversity despite the challenges faced by raising a child with ASD [34,35,36].

This is also partially confirmed by research conducted by Freisen et al. (2021) during the COVID-19 pandemic among caregivers of children with ASD. They show that moderate resilience was achieved by 32.6% of the studied caregivers of children with ASD during the COVID-19 pandemic (the second largest group), low resilience in 48.9% of respondents, and high resilience in only 18.5%. The research shows that this level has not changed significantly and is moderate in a large group of respondents, which can be viewed with optimism and the belief that parents have the inner strength to overcome adversity [37]. Research has revealed that resilient people learn to cope with stressful situations by adapting to experienced threats and traumatic experiences. There are no statistically significant differences in the style of coping with stress between the group of wives and their husbands. The results are in the range of average results, and both studied groups show a moderate tendency to use, first of all, the technique of problem-solving by cognitive reformulation of the acquired information, which they can also use to plan problem-solving (Task-oriented coping (SSZ)). The remedial activity of the spouses studied is adjusted to the changing needs of the child with ASD, but also to changes in the situation related to the pandemic and the related limitations and threats. The controllable and uncontrollable random elements of both the pandemic and caring for a disabled child force the spouses to make changes in their preferred coping style. Therefore, in such a difficult situation, the results they obtained, indicating a moderate level of using the avoidance style of coping with stress, are not surprising. Women prefer to seek social contacts, while men engage in substitute activities. Refraining from thinking about and acting on the problem protects the spouses from excessive fear, despair and hopelessness. It allows to distract attention from a dramatic parenting situation in which the prognosis for the development of a child with ASD is uncertain. It can be concluded that the avoidance style of coping with stress enables the studied wives and husbands to prepare personal resources to deal with the problem through active, task-oriented coping with caring for a child with ASD during the COVID-19 pandemic.

The general assessment result obtained (4 sten- low result) on the quality of the marital relationship in the group of wives and husbands indicates a lack of satisfaction with the marital relationship. However, some of the results of the individual dimensions on the scale oscillate between 4 and 7 sten, indicating low and moderate satisfaction with marriage. In terms of the scales of intimacy, self-realization and similarity, no significant statistical differences were found between the spouses, which proves they have a similar perception of their relationship. It is worth noting that husbands rated the dimensions of intimacy, disappointment and general satisfaction with the relationship slightly lower than their wives, but rated self-realization and similarity higher. There was one significant difference between the studied wives and husbands raising a child with ASD in terms of disappointment with marriage, which was high in the wives and average in the husbands. The wives probably feel more overburdened by caring for a child with ASD, and more often they may think of their relationship in the category of a sense of life failure, where independence and personal autonomy are disturbed. The more so because the wives obtained the lowest result in self-realization, which may indicate that the relationship and raising of a child with ASD and the required therapy, to some extent prevent them from realizing themselves, their system of values and their life tasks. In the remaining dimensions, the respondents obtained similar results, which proves a similar assessment of the quality of the marriage relationship. The low result in overall marital satisfaction in this study is puzzling, as spouses have resources that protect them from stress. Perhaps the overall level of satisfaction with the relationship is lower, but its individual factors are average, which shows that they accept things as they are, and cope with the emotional, cognitive and behavioural problems associated with caring for a child with ASD during the pandemic despite disappointment and a willingness to make their relationship look different. They appreciate intimacy and closeness, empathy, relationship with a life partner and cooperation with them that can keep up their spirits and overcome the problems of a changed everyday life, as evidenced by the results on the average level of intimacy and similarity.

A comparative analysis of the average results obtained in the individual areas studied did not show any statistically significant differences. In fact, research conducted among parents of children with ASD before the pandemic shows differences in the preferred styles of coping with stress. The results of the research and analyses by E. Pisuli (2002) show that parents more often use the avoidance style of coping with stress [38]. Research by Sekułowicz (2013) showed that mothers of children with autism spectrum disorders used different coping strategies than fathers. High and continuous stress in mothers also increased the risk of burnout. The coping strategies used include seeking emotional support, discharging emotions and denial, and in the case of mothers who are most burned out, ceasing activities. Fathers declared that they use positive re-evaluation, and they are also more satisfied with the support received than women. In times of family crisis, mothers cope worse and use emotions and avoidance behaviours [39]. In a similar study by M. Dudek (2017), among parents of children with ASD, mothers relatively more often than fathers display the avoidance-focused style (SSU) and the style of engaging in substitute activities (ACZ) [40].

In this study, it is only the mosaic of dependencies between their properties (traits) that shows the differences between men and women raising a child with ASD during the pandemic. Particularly marked differences appear in the relationship between resilience and the coping styles of wives and husbands. In the group of women, persistence, openness, personal competence and tolerance towards failure increase the level of the task-oriented style of coping with stress. In the group of men, only persistence and an optimistic attitude to life correlate directly with the task-oriented style of coping with difficult situations. In both women and men, all resilience factors negatively correlate with the emotional coping style. However, both husbands and wives use resilience for the avoidance coping style of raising a child with ASD. Women look for social contacts and men engage in various alternative activities. The picture of the indicated relationships between the studied variables shows that both wives and husbands use their resilience to deal with the traumatic situation of raising a child with ASD, especially during the COVID-19 pandemic, while protecting themselves against despair, fear and hopelessness through the use of unique coping styles. This is confirmed by previous pre-pandemic studies conducted by Byra and Parchomiuk (2015) among mothers of children with ASD, where a significant but weak relationship between resilience and strategies was found. Positive relationships between selected resilience indices and task coping, and negative relationships with emotional-avoidance were established [41]. Other research by Gagat-Matuła (2021) conducted during the COVID-19 pandemic among parents of children with ASD showed that respondents more often prefer constructive coping with stress using the task focused style (SSZ) if a spouse assesses both themselves and their spouse as more supportive and engaged in communication (marriage communication) and less deprecating. Moreover, the greater the difference between self-assessment and that of the spouse in deprecating communication (marriage communication), the less often the task-focused style (SSZ) was preferred. The results are statistically significant [42].

Another area showing the differences between wives and husbands is the relationship between their psychological resources and the assessment of marital satisfaction, calculated using backward stepwise regression analysis. Openness in both studied groups is a significant predictor of intimacy, allowing spouses to feel close to each other and to feel emotional contact in difficult life situations. Perseverance is the predictor of self-fulfilment in the relationship for both wives and husbands. In the case of women, this is also true for an optimistic attitude to life, and in men, the avoidance style—engaging in alternative activities (ACZ).

Wives and husbands differ in terms of predictors of similarity and disappointment. In women, openness and an optimistic attitude to life have a significant positive impact on the aforementioned factor of marital satisfaction, that is similarity. In turn, in men, persistence has a significant positive effect on this factor. The predictor of disappointment in women is the avoidance style—engaging in alternative activities (ACZ), and in men the emotion-oriented coping style (SSE). This proves that these styles act directly in proportion to the factor of disappointment in the assessment of marital satisfaction. This result shows that disappointment in their relationship increased in women raising a child with ASD during the pandemic. Meanwhile, engaging in alternative activities (watching TV, video games, getting enough sleep, wishful thinking about pleasure, sometimes overeating) gives them the opportunity to reduce emotional tension and prevents them constantly thinking about parental trauma, although unfortunately this translates into disappointment with marriage. In turn, in men, the predictor of disappointment in a relationship is the emotion-oriented coping style (SSE), which consists of experiencing and trying to discharge emotions. This can involve worrying about things, complaining or fantasizing, or using “it’s going to be all right” wishful thinking without making any particular effort to make it work. Failure to make an attempt increases disappointment in the relationship over time. It is worth noting that the preferred avoidance styles limit the spouses’ time together and may lower their satisfaction with their marriage. Interesting results were found in research conducted by Bijing He et. al. (2021) during the pandemic among a large population of married couples with a child with ASD. These show that the time spent raising children had a negative impact on the quality of the relationship because it directly limited the time parents could devote to their personal relationship. The longer the time spent by the wives had no effect on their relationship with their husbands; in fact, the time wives spent on their children also made their husbands dissatisfied. This can be explained by the fact that the husbands were unhappy because the wives spent so much time with their children, resulting in husbands feeling neglected [43]. Also, the Gagat-Matuła (2021) study conducted in the COVID-19 pandemic among married couples with a child with ASD, showed that spouses more often prefer non-constructive coping with stress using the emotion-focused style (SSE) or the avoidance-focused style (SSU) if the spouses assesses both themselves and their spouse as depressed. The greater the difference between their own assessment and the assessment of their spouse in supportive communication, the more often the respondents prefer the style focused on emotions (SSE) and the style focused on avoidance (SSU) [43].

This study has some advantages and, at the same time, interpretation limitations. One of the limitations is the targeted selection of groups of spouses caring for a child with ASD (selection based on the medical diagnosis of the child), while at the same time this is an advantage because the selection took place during the pandemic (access to the sample was significantly limited) and the research was not conducted online. Where we were not sure if the respondents were the parents of a child with ASD, we had such certainty because the research was carried out at the Specialist Clinic for People with Autism. The study also included only married couples with children aged 5–8 years old with level 3 autism spectrum disorder (according to the DSM-5), while children with ASD at levels 1 and 2 and at other stages of the child’s development were not included. The analyses have not been redrawn with the use of sociodemographic variables, which may turn out to be important, so they will be used in further analyses of the above-mentioned studies. The sociodemographic data in this article served to illustrate the research group. It was worth extending and comparing the results of the study to a control group of married couples without a child with ASD, especially since the COVID-19 pandemic did not only affect the parents of children with ASD. It may be interesting to compare these results. It was also worth broadening the survey to include questions about social behavior that contributed to such marriage problems during the pandemic. The results of longitudinal studies conducted before, during and after the COVID-19 pandemic could be of interest. Despite the presented limitations, the results of the study bring new content to the issues of marital satisfaction of parents who care for a child with ASD in a period of special limitations and threats such as the COVID-19 pandemic, especially since there are few studies conducted in this area.

## 5. Conclusions

The conducted comparative analysis of the average results obtained by the studied groups in terms of resilience and styles of coping with stress did not show any significant statistical differences, and the results of their dimensions were at a moderate level. There was no statistically significant difference between women and men in the general level of marital satisfaction, which turned out to be low, although the level of the individual dimensions of intimacy, self-fulfilment and similarity was moderate, and the level of disappointment was high. Wives feel much more disappointed with their marriage than husbands, which is perhaps related to the difficulty of raising a child with ASD in such an unfavourable period as the COVID-19 pandemic. In the group of wives, the following resilience factors increase their assessment of marital satisfaction: perseverance, openness, personal competence, and tolerance of failure increase the level of the task-oriented style of coping with stress. In the group of husbands, persistence and an optimistic attitude to life increase the level of the task-oriented style of coping with difficult situations. It is worth emphasizing that in both women and men, all resilience factors negatively correlate with the emotional coping style. Although husbands and wives also use resilience to avoid the coping style of raising a child with ASD, women look for social contacts and men engage in various alternative activities. The aforementioned relationships show that both wives and husbands use their resilience for task-oriented coping with the traumatic situation of raising a child with ASD, especially during the COVID-19 pandemic, and at the same time protect themselves against despair, fear, burnout and powerlessness through the use of an avoidance coping style. In both the studied groups, openness is a significant predictor of intimacy, allowing spouses to feel close to each other and to feel emotional contact in difficult life situations. Perseverance is the predictor of self-fulfilment in the relationship for both wives and husbands. In the case of women, this is also true for an optimistic attitude to life, while in men, for the avoidance style—engaging in alternative activities (ACZ). Wives and husbands differ in the predictors of similarity and disappointment. In women, openness and an optimistic attitude to life have a positive effect on similarity, while in men, persistence has a significant positive effect on this factor. The predictor of disappointment in women is avoidance-oriented coping (ACZ), and in men—emotion-oriented coping (SSE). This result shows that disappointment with the relationship increased in women in the situation of raising a child with ASD during the pandemic. Meanwhile, engaging in alternative activities gives them the opportunity to reduce emotional tension and avoid constantly thinking about everyday problems, although unfortunately this translates into disappointment with their marriage. In turn, in men, the predictor of disappointment with a relationship is the emotion-oriented coping style (SSE), which consists of worrying about various matters without making any specific efforts to resolve them. Failure to make an attempt increases disappointment with the relationship over time.

The results indicate the need to support married couples raising children with ASD during periods of isolation. Intervention and social support are needed so that spouses can draw on personal resources that translate into overall satisfaction with their marriage, thus easing the hardships associated with care for and therapy of a child with ASD.

The Ethics Committee of the Scientific Research Faculty at the Pedagogical University of Cracow has reviewed and approved the research. Document WP.111-6.19.

## Figures and Tables

**Table 1:** Comparative analysis of resilience, stress coping styles and marital satisfaction among wives and husbands.

Variable	Wives		Husbands		T	*p*
	M	SD	M	SD		
**SPP-25**						
General resilience	69.19	15.99	71.02	11.90	−0.971	0.311
Perseverance	14.83		14.33	2.91	0.611	0.555
Openness	15.11	3.19	15.31	2.64	−0.630	0.521
Competence	13.47	3.88	14.51	2.93	−1.523	0.127
Tolerance	13.49	3.71	14.59	2.51	−1.451	0.177
Optimistic attitude	12.29	4.01	12.28	2.77	−1.311	0.184
**CISS**						
Task-oriented coping (SSZ)	57.01	9.33	58.05	7.01	−1.433	0. 144
Emotion-oriented coping (SSE)	44.03	9.88	42.06	9.73	0.966	0.311
Avoidance-oriented coping (SSU)	42.77	7.77	41.02	7.05	1.399	0.162
Engaging in substitute activities (ACZ)	18.80	5.04	17.99	4.52	0.701	0.499
Seeking social contacts (PKT)	17.01	3.59	15.05	3.33	1.799	0.077
**Well-Matched Marriage Questionnaire (KDM-2)**						
Intimacy	28.11	6.13	27.43	5.81	1.888	0.182
Personal Fulfillment	24.06	4.93	25.21	5.07	1.321	0.092
Similarity	24.88	5.02	25.11	5.05	1.762	0.087
Disappointment	26.97	6.35	24.37	4.88	2.384 *	0.021
General result	104.02	23.11	102.12	21.06	0.986	0.439

* *p* < 0.05. Source: own study.

**Table 2:** Pearson’s r correlation coefficients between resilience and stress coping styles among the study group of wives and husbands.

SPP-25	Research Group	Task-Oriented Coping (SSZ)	Emotion-Oriented Coping (SSE)	Avoidance-Oriented Coping (SSU)	Engaging in Substitute Activities (ACZ)	Seeking Social Contacts (PKT)
General resilience	Wives	0.444 ***	−0.398 **	0.215	0.070	0.397 **
	Husbands	0.468 **	−0.479 **	−0.038	−0.173	0.167
Perseverance	Wives	0.399 **	−0.099	0.224	−0.049	0.290
	Husbands	0.438 **	−0.267	−0.167	0.399 *	−0.077
Openness	Wives	0.440 ***	−0.378 **	0.179	−0.088	0.391 *
	Husbands	0.247	−0.187	0.078	−0.041	0.271
Competence	Wives	0.399 **	−0.469 **	0.241	0.069	0.298 *
	Husbands	0.206	−0.448 **	0.027	−0.119	0.155
Tolerance	Wives	0.399 **	−0.389 **	0.401 **	0.133	0.555 ***
	Husbands	0.301	−0.477 **	−0.058	−0.077	0.094
Optimistic attitude	Wives	0.253	−0.459 ***	0.261	0.135	0.326 *
	Husbands	−0.489 **	−0.155	−0.237	0.051	0.062

* *p* < 0.05; ** *p* < 0.01; *** *p* < 0.001. Source: own study.

**Table 3:** Predictors (resilience, stress coping styles) of marital satisfaction among the studied wives.

Explanatory Variable (Indicators for the Whole Regression Model)	Explanatory Variable	*R*2	b	B	SEB	F	*p*
Intimacy (*R* = 0.378. *R*2= 0.143)	Openness	0.378	0.141	1.63	0.599	2.703	0.011
	‘y-intercept’			58.30	9.308	6.265	0.001
Personal Fulfillment (*R* = 0.590. *R*2 = 0.349)	Perseverance	0.589	0.211	2.30	0.819	2.81	0.009
	Optimistic attitude	−0.467	0.181	−1.70	0.656	−2.60	0.014
	‘y-intercept’			48.52	9.271	5.24	0.001
Similarity (*R* = 0.520. *R*2 = 0.267)	Openness	0.557	0.224	2.21	0.89	2.50	0.018
	Optimistic attitude	−0.475	0.192	−1.77	0.72	−2.50	0.018
	Wyraz wolny			55.87	10.03	5.59	0.001
Disappointment (*R* = 0.572. *R*2 = 0.327)	Engaging in substitute activities (ACZ)	0.58 5	0.143	0.66	0.30	2.224	0.033
	‘y-intercept’			37.42	10.97	3.416	0.002
Wynik ogólny (*R* = 0.550. *R*2 = 0.302)	Openness	0.570	0.219	6.5	2.46	2.62	0.011
	‘y-intercept’			162.7	27.81	5.86	0.001

*R*—multiple correlation coefficient value, *R*2—variant coefficient explained by explanatory variables, b—standardised regression coefficient, B—regression coefficient, SEB—standard estimate error.

**Table 4:** Predictors (resilience, stress coping styles) of marital satisfaction among the studied husbands.

Explanatory Variable (Indicators for the Whole Regression Model)	Explanatory Variable	*R*2	b	B	SEB	F	*p*
Intimacy (*R* = 0.566. *R*2 = 0.322)	Openness	0.530	0.144	1.84	0.50	3.71	0.001
	‘y-intercept’			54.21	11.57	4.67	0.001
Personal Fulfillment (*R* = 0.574. *R*2 = 0.328)	Perseverance	0.586	0.143	2.14	0.53	4.118	0.001
	Engaging in substitute activities(ACZ)	0.317	0.144	0.67	0.30	2.224	0.033
	‘y-intercept’			38.42	10.97	3.416	0.002
Similarity (*R* = 0.471. *R*2 = 0.223)	Perseverance	0.471	0.145	1.52	0.490	3.279	0.002
	‘y-intercept’			62.17	7.270	9.552	0.001
Disappointment (*R* = 0.499. *R*2 = 0.333)	Emotion-oriented coping (SSE)	0.299	0.145	1.6	0.80	2.085	0.004
	Wyraz wolny			52.13	11.44	3.017	0.002
General result (*R* = 0.568. *R*2 = 0.322)	Perseverance	0.583	0.142	5.7	1.40	4.088	0.002
	‘y-intercept’			134.8	29.47	4.568	0.001

*R*—multiple correlation coefficient value, *R*2—variant coefficient explained by explanatory variables, b—standardised regression coefficient, B—regression coefficient, SEB—standard estimate error.

## Data Availability

The quantitative that support the findings of this study are available on request from the corresponding author. The data are not publicly available due to privacy and ethical or legal restrictions.
